# Neoadjuvant chemotherapy affects molecular classification of colorectal tumors

**DOI:** 10.1038/oncsis.2017.48

**Published:** 2017-07-10

**Authors:** K Trumpi, I Ubink, A Trinh, M Djafarihamedani, J M Jongen, K M Govaert, S G Elias, S R van Hooff, J P Medema, M M Lacle, L Vermeulen, I H M Borel Rinkes, O Kranenburg

**Affiliations:** 1 Department of Surgical Oncology, UMC Utrecht Cancer Center, University Medical Center, Utrecht, The Netherlands; 2 Department of Medical Oncology, Dana-Farber Cancer Institute, Boston, MA, USA; 3 Julius Center for Health Sciences and Primary Care, University Medical Center, Utrecht, The Netherlands; 4 Laboratory for Experimental Oncology and Radiobiology (LEXOR), Center for Experimental Molecular Medicine (CEMM), Academic Medical Center, University of Amsterdam, Amsterdam, The Netherlands; 5 Department of Pathology, UMC Utrecht Cancer Center, University Medical Center, Utrecht, The Netherlands

## Abstract

The recent discovery of ‘molecular subtypes’ in human primary colorectal cancer has revealed correlations between subtype, propensity to metastasize and response to therapy. It is currently not known whether the molecular tumor subtype is maintained after distant spread. If this is the case, molecular subtyping of the primary tumor could guide subtype-targeted therapy of metastatic disease. In this study, we classified paired samples of primary colorectal carcinomas and their corresponding liver metastases (*n*=129) as epithelial-like or mesenchymal-like, using a recently developed immunohistochemistry-based classification tool. We observed considerable discordance (45%) in the classification of primary tumors and their liver metastases. Discordant classification was significantly associated with the use of neoadjuvant chemotherapy. Furthermore, gene expression analysis of chemotherapy-exposed versus chemotherapy naive liver metastases revealed expression of a mesenchymal program in pre-treated tumors. To explore whether chemotherapy could cause gene expression changes influencing molecular subtyping, we exposed patient-derived colonospheres to six short cycles of 5-fluorouracil. Gene expression profiling and signature enrichment analysis subsequently revealed that the expression of signatures identifying mesenchymal-like tumors was strongly increased in chemotherapy-exposed tumor cultures. Unsupervised clustering of large cohorts of human colon tumors with the chemotherapy-induced gene expression program identified a poor prognosis mesenchymal-like subgroup. We conclude that neoadjuvant chemotherapy induces a mesenchymal phenotype in residual tumor cells and that this may influence the molecular classification of colorectal tumors.

## Introduction

Colorectal cancer (CRC) is one of the leading causes of cancer-related mortality. The vast majority of CRC patients die from metastatic disease. At the time of presentation 20–25% of CRC patients already have metastatic disease and an additional ~35% will develop metastases during follow-up.^1, 2, 3^

The currently used clinical and pathological parameters have insufficient predictive power to identify the patients who are at risk of developing metastases. Recently, gene expression-based molecular classification studies have identified subgroups of human colon cancer.^4, 5, 6, 7, 8, 9, 10, 11^ Cross-comparison of these studies subsequently revealed the existence of four consensus molecular subtypes (CMS), which are characterized by differential activity of various signaling pathways.^12^ Interestingly, the molecular subtypes also differ in terms of prognosis. Notably, tumors of the ‘mesenchymal-like’ subtype (CMS4) have a shorter disease-free and overall survival due to higher metastatic potential and relative resistance to chemotherapy- and EGFR-targeted therapy.^12, 13, 14^ To enable implementation of the novel classification system in clinical practice, we recently developed an immunohistochemistry-based diagnostic test that distinguishes mesenchymal-like cancers from epithelial-like subtypes based on the expression of five markers in tumor cells (HTR2B, FRMD6, ZEB1, CDX2 and pancytokeratin).^13^ This test can be used for patient stratification and the development of subtype-directed therapies.

The molecular classification system and the novel diagnostic test are based on the analysis of primary colorectal tumors. It is currently unknown whether molecular subtypes are preserved in metastatic cancer, and whether classification of primary colorectal tumors can guide subtype-targeted therapy for metastatic disease. Dissemination and homing to a different organ environment requires adaptations by cancer cells to survive and proliferate, which could result in altered gene expression patterns. Moreover, neoadjuvant therapy is frequently used to downsize primary (rectal) tumors and liver metastases, and may change the constitution of the tumor bulk and affect gene expression in residual cancer cells.

## Results and discussion

### Neoadjuvant therapy of primary colorectal tumors is associated with a mesenchymal tumor subtype

**Figure 1:**
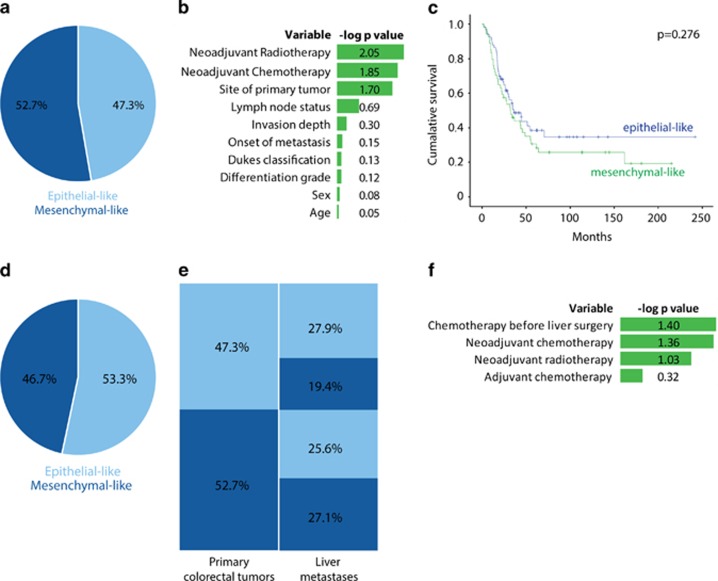
Discordant classification of primary colorectal tumors and corresponding liver metastases. (**a**) The tissue microarray (TMA) was constructed from the resection specimens of primary colorectal tumors and the resection specimens of colorectal liver metastases of 129 patients. Tumor-rich areas were identified via haemotoxylin and eosin stainings and three cores of 0.6 mm were obtained per tumor type. Digital images of TMA immunohistochemically stained slides were obtained via an Aperio Scanscope XT system (Leica Biosystems, Wetzlar, Germany). These were automatically analyzed as described before.^13^ Cores with a random forest probability of 60% were scored as 'mesenchymal-like'. Patient subtypes were determined using majority consensus. Here a pie chart shows the distribution of epithelial-like and mesenchymal-like of the primary colorectal tumors in our paired tumor cohort. (**b**) Patient characteristics of epithelial-like tumors were compared to mesenchymal-like tumors. Age was compared via the Wilcoxon rank sum test, for all other variables the *X*^2^-test was used. Minus log10 *P*-values were calculated and are shown in the graph. Table 1 shows the detailed list of patient characteristics. (**c**) Kaplan–Meier survival curves of the overall survival after liver resection, calculated with a log-rank test (*P*=0.276). The blue line represents the patients with epithelial-like colorectal tumors and the green line represents patients with mesenchymal-like colorectal tumors. (**d**) Pie chart of the classification of liver metastases of our paired tumor cohort. (**e**) Relationship between the classification of primary colorectal tumors and the corresponding liver metastases. (**f**) The influence of chemotherapy on the classification of paired tumors. Neoadjuvant chemotherapy for the primary colorectal tumors or liver metastases and adjuvant therapy for the primary CRC were all univariate analyzed via the *X*^2^-test. Chemotherapy was considered neoadjuvant if it was given to downsize the tumor, primary or metastases, prior to the surgery. Adjuvant chemotherapy is chemotherapy given after the initial resection of the primary colorectal tumor. Concordant tumor pairs were depicted to discordant tumor pairs, which were separated in switching from epithelial-like to mesenchymal-like and vice versa. Minus log10 *P*-values were calculated and depicted in this graph.

Paraffin-embedded tissue samples of the resection specimens of paired primary colorectal tumors and corresponding liver metastases were available of 129 patients, and were assembled into a tissue microarray. This cohort solely consists of patients with operable colorectal liver metastases. The tissue microarray is created of the resection specimens of both tumors of these patients. All patient characteristics are described in Table 1. In brief, the majority was male, had synchronous metastases and a moderately differentiated colon tumor. Tissue microarray sections were subsequently used for analysis of the expression of HTR2B, FRMD6, ZEB1, CDX2 and pancytokeratin by immunohistochemistry, as described previously.^13^ After digital analysis of the staining patterns of the primary tumors, 61 tumors were scored as epithelial-like (47.3%) and 68 were scored as mesenchymal-like (52.7%) (Figure 1a). In the current cohort—consisting exclusively of metastasized tumors—the percentage of mesenchymal-like tumors is approximately two-fold higher compared to studies on stage I–IV primary colorectal tumors.^12^ This is in line with previous analyses of the mesenchymal phenotype in two large cohorts of metastasized primary colorectal tumors.^13, 15, 16^ Mesenchymal-like tumors have a higher risk of recurrence, which explains their enrichment in these cohorts.

Patient and tumor characteristics (including age, gender, onset of disease, invasion depth, lymph node status, Dukes classification and differentiation status) were equally distributed between the two groups of patients with epithelial-like and mesenchymal-like tumors. However, univariate analysis showed differences in tumor localization: mesenchymal-like tumors were more often located in the rectum, whereas epithelial-like tumors were predominantly located in the sigmoid (*P*=0.020). Furthermore, we found that patients with mesenchymal-like tumors had more frequently received neoadjuvant radiotherapy (*P*=0.009) and neoadjuvant chemotherapy (*P*=0.013) compared to patients with epithelial-like tumors (Figure 1b; Table 1). Multivariate analysis identified neoadjuvant chemotherapy as an independent predictor (*P*=0.012).

As neoadjuvant chemoradiation is mainly administered to patients with rectal cancer, tumor localization could be a confounding factor in the association between chemoradiation and the mesenchymal phenotype. However, rectal cancers treated with neoadjuvant chemo- and/or radiotherapy more often had a mesenchymal phenotype than untreated rectal cancers (71% versus 30%, respectively; *P*=0.027), indicating that neoadjuvant therapy is a predictor of the mesenchymal phenotype, independent of tumor localization. These findings are in line with previous results showing that post-treatment rectal tumors were mostly classified to the stroma-rich subtype,^17^ although this classification was based on stromal parameters.

Mesenchymal-like colorectal tumors (CMS4) have a poorer prognosis compared to the other three CMS.^12^ In this cohort, which consists solely of patients with operable metastatic colorectal tumors, a trend of survival disadvantage for mesenchymal-like tumors could be observed (*P*=0.276, Figure 1c). The hazard ratio after multivariate analysis is 1.441 (95% confidence interval (CI) 0.893–2.325, *P*=0.134).

### Discordant molecular classification of primary colorectal tumors and their corresponding liver metastases is associated with neoadjuvant chemotherapy

The immunohistochemical diagnostic test was also applied to the liver metastases of the same patients: 69 metastases were scored as epithelial-like (53.3%) and 60 metastases were scored as mesenchymal-like (46.5%), which roughly corresponds to the subtype distribution of the primary tumors (Figure 1d). However, the classification of primary tumors and the corresponding liver metastases was concordant in only 71 pairs (55% (95% CI 46.3–63.7)), of which 36 were epithelial-like tumors (27.9% (95% CI 20.1–35.8)) and 35 were mesenchymal-like tumors (27.1% (95% CI 19.4–34.9)). Of the 58 discordant tumor pairs (45% (95% CI 36.3–53.7)), 25 primary colorectal tumors that were classified as epithelial-like (19.4% (95% CI 12.5–26.3)) gave rise to liver metastases that were classified as mesenchymal-like, and 33 mesenchymal-like colorectal tumors (25.6% (95% CI 18.0–33.2)) gave rise to epithelial-like liver metastases (Figure 1e). This frequently discordant molecular classification is in contrast with the high concordance that has been reported for mutations in driver genes in primary tumors and the corresponding metastases, for example, *KRAS*, *BRAF* and *PIK3CA*.^18, 19^ This finding is important as it indicates that molecular subtyping via immunohistochemistry of primary colorectal tumors cannot simply be extrapolated to classify metastatic disease.

We observed a significant correlation between the administration of chemotherapy prior to tumor resection (neoadjuvant chemotherapy) and discordant classification. More than half of the primary tumors that were exposed to neoadjuvant chemotherapy showed discordant classification of the tumor pairs. In the majority of the cases, this was a switch from a mesenchymal-like primary colorectal tumor, to an epithelial-like liver metastasis, rather than vice versa (*P*=0.044). Similarly, discordant classification was more common in patients who received chemotherapy prior to resection of liver metastasis compared to those who did not. In these cases a switch from an epithelial-like primary colorectal tumor (not exposed to chemotherapy) to a mesenchymal-like liver metastasis (exposed to chemotherapy) was predominant (*P*=0.040) (Figure 1f; Supplementary Table 1). These findings suggest that neoadjuvant chemotherapy influences cancer cell biology and drives colorectal tumors toward a more mesenchymal-like phenotype.

Molecular changes in response to chemotherapy have been described for other types of cancer. For example, neoadjuvant treatment in breast cancer is associated with gene expression changes and discordant molecular subtype classification in 38% of the cases.^20, 21^ Chemotherapy-induced changes in HER2 status are also frequently observed in breast cancer and have a potential impact on clinical management.^22^ Besides chemotherapy, the molecular subtype could also be influenced by the organ microenvironment and/or intra-tumor heterogeneity, and/or heterogeneity between distinct metastases. Indeed, we have recently found that there is extensive subtype heterogeneity among different regions of the same tumor in approximately half of the colorectal tumors analyzed.^23^ When selecting patients for subtype-targeted therapies, all factors influencing the tumor subtype should be taken into consideration.

### Chemotherapy-surviving tumor cells express a mesenchymal gene signature that identifies aggressive CMS4-like tumors.

**Figure 2:**
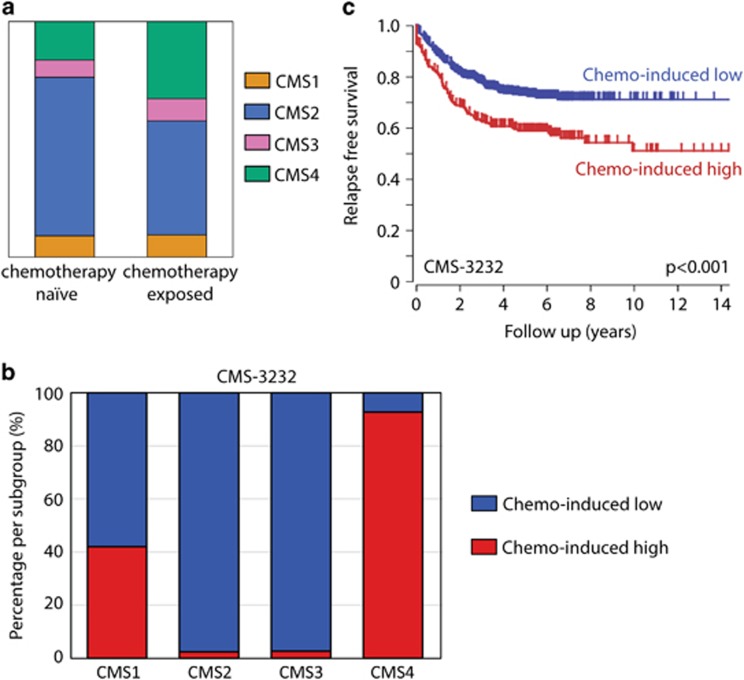
5-FU-based chemotherapy is associated with a mesenchymal tumor phenotype. (**a**) In the liver metastases data set two groups were made, chemotherapy naive and chemotherapy exposed, these were compared in distribution of the CMS classification. The bar graph shows chemotherapy before surgery is associated with an increased proportion of mesenchymal-type tumors (CMS4). The CMS subgroups are CMS1: orange, CMS2: blue, CMS3 pink, CMS4: green. (**b**) The chemotherapy-induced genes were used to cluster the tumors of the CMS cohorts into chemo-induced high and low subgroups (K-means option in R2, using a two group separation) based on single gene *P*-values. All tumors had previously been classified into molecular subtypes. The graphs show the distribution of the CMS subtypes within the chemo-induced high and low subgroups. The chemo-induced high subgroup is enriched for mesenchymal subtypes (CMS4). (**c**) Kaplan–Meier curves showing the differences in relapse-free survival between the chemo-induced high and low subgroups in the CMS-3232 cohort.

To further explore the relationship between neoadjuvant therapy and the mesenchymal phenotype, we classified liver metastases from a previously published data set^24^ with the CMS classifier.^5, 12^ In this same set of liver metastases, we compared chemotherapy-exposed liver metastases to (*n*=64) chemotherapy naive liver metastases (*n*=55). Chemotherapy-exposed metastases were more frequently classified as mesenchymal-like compared to the chemotherapy naive liver metastases (33% versus 16%, *P*=0.06, Figure 2a). In comparison, ~32% of liver metastases exposed to prior treatment in the Khambata-Ford *et al.*^25^ cohort were classified as the mesenchymal subtype.^5^ In an unbiased approach, we identified all genes that were higher expressed in chemotherapy-exposed liver metastases compared to chemotherapy naive liver metastases. These genes were used to cluster the primary tumors of two large cohorts into high and low subgroups. The tumor group expressing high levels of these genes was strongly enriched for mesenchymal-type tumors (CMS4) in the CMS-3232 cohort^12^ (Figure 2b). These patients had a significantly reduced relapse-free survival probability (Figure 2c).

**Figure 3:**
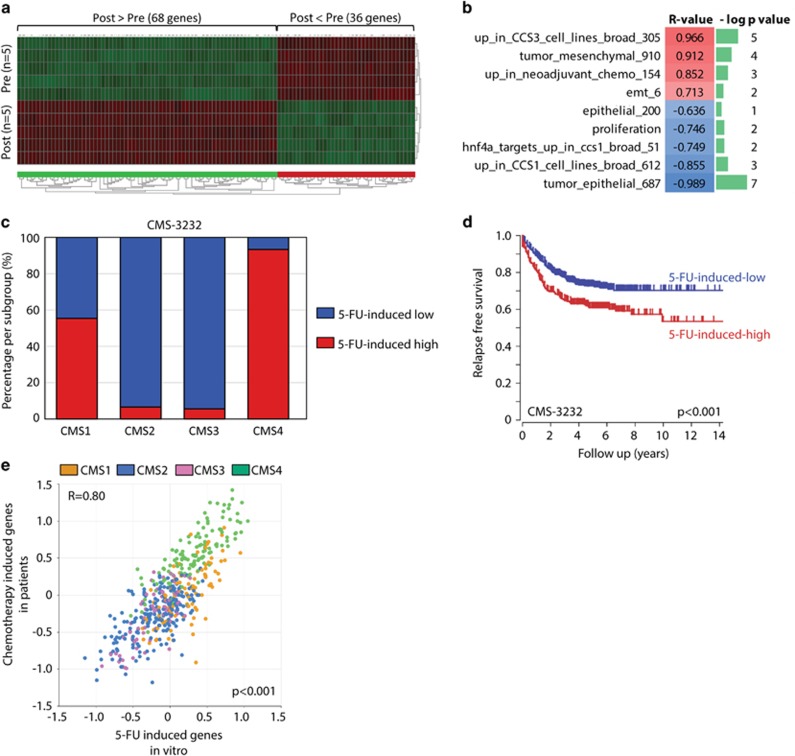
Chemotherapy induces mesenchymal gene expression in patient-derived colonospheres. (**a**) Liver metastasis-derived colonospheres were treated with 5-FU for six cycles. RNA was isolated from control (*n*=5) and 5-FU-treated cells (*n*=5), and were analyzed by gene expression profiling. The heat map shows all genes that were significantly (*P*<e^−6^) upregulated (68) or downregulated (36) in post-treatment tumor cells. See Supplementary Table 2 for a full list of genes. (**b**) Expression of the 5-FU-induced gene set (68 genes; Supplementary Table 2) was correlated with gene sets reflecting either an epithelial or a mesenchymal tumor phenotype in the data set of the same experiment. Correlations were assessed by using the ‘gene set versus gene sets’ option in the R2 genomics analysis and visualization platform. Gene sets reflecting a mesenchymal tumor cell phenotype positively correlate with the 5-FU-induced gene set (indicated in red), while gene sets reflecting an epithelial phenotype show a negative correlation (in blue). The *P*-values of the correlations are indicated as minus log10 *P*-values in green bars. (**c**) The 68 5-FU-induced genes were used to cluster the tumors of the CMS cohorts into 5-FU-induced high and low subgroups (K-means option in R2, using a two group separation) based on single gene *P*-values. All tumors had previously been classified into molecular subtypes. The graphs show the distribution of the CMS subtypes within the 5-FU-induced high and low subgroups. The 5-FU-induced high subgroup is enriched for mesenchymal subtypes (CMS4). (**d**) Kaplan–Meier curves showing the differences in relapse-free survival between the 5-FU-high and 5-FU-low subgroups in both cohorts. (**e**) Expression of the experimental-derived gene set of 5-FU-induced genes was compared to expression of genes upregulated in chemotherapy-exposed liver metastases by using the ‘relate 2 tracks’ option in the R2 genomics analysis and visualization platform. The XY-plot shows the correlation of the expression of both gene sets (*R*=0.80, *P*=2.0e^−110^) in the CIT subset of the CMS cohort. The CMS subgroups are CMS1: orange, CMS2: blue, CMS3 pink, CMS4: green.

The vast majority of patients receiving neoadjuvant therapy prior to primary tumor resection are patients with rectal cancer. According to the Dutch guidelines, neoadjuvant chemotherapy in this patient category consists of oral capecitabine, a 5-fluorouracil (5-FU) prodrug, in combination with radiation therapy.^26, 27^ To study a potential causal relationship between chemotherapy and mesenchymal gene expression we exposed patient-derived colonospheres to six cycles of 5-FU. RNA was isolated from colonospheres prior to treatment (*n*=5) and from the surviving tumor cells after the last cycle (*n*=5). Gene expression profiling revealed that 5-FU treatment resulted in drastic changes in gene expression (Supplementary Table 2), with 68 significantly upregulated genes and 36 significantly downregulated genes (*P*<e^−6^; Figure 3a). Additionally, we found that expression of the 68 5-FU-induced genes was strongly correlated with the expression of previously published gene sets reflecting a mesenchymal tumor phenotype. These include (i) the core drivers of epithelia–mesenchymal transition, (ii) genes that are upregulated in liver metastases that have previously been exposed to chemotherapy, (iii) genes that are highly expressed in the tumor cell compartment of mesenchymal-like tumors^28^ and (iv) genes that are highly expressed in mesenchymal-like colorectal cancer cell lines *in vitro*.^28^ By contrast, the 68 5-FU-induced genes showed a strong negative correlation with (i) a gene set reflecting epithelial differentiation,^28^ (ii) genes that are highly expressed in the tumor cell compartment of epithelial-like tumors,^28^ (iii) genes that are highly expressed in epithelial-like colorectal cancer cell lines *in vitro* and (iv) the target genes of HNF4a, a master inducer of epithelial differentiation and suppressor of mesenchymal genes^28^ (Figure 3b).

To address this further, we used the 68 5-FU-induced genes to cluster the primary colorectal tumors of two large cohorts into two subgroups with high and low expression of the 5-FU-induced genes (K-means). The 5-FU-high groups were strongly enriched for mesenchymal-type tumors (CMS4) in the CMS-3232 cohort^12^ (Figure 3c) and had a significantly reduced relapse-free survival probability (Figure 3d). To further assess the relevance of the 5-FU-induced gene set derived from the *in vitro* experiments, we compared its expression to the genes that are upregulated in liver metastases of patients who were exposed to neoadjuvant chemotherapy (Supplementary Table 3). This revealed a strong correlation (*R*=0.80, *P*=2.0e^−110^) between the expression of both independently generated gene sets (Figure 3e). The tumors expressing the highest levels of 5-FU-induced genes (both the *in vitro*-derived and patient-derived signatures) were enriched in CMS4 (right upper quadrant), indicating that chemotherapy-induced genes are already highly expressed in treatment-naive mesenchymal-like primary colorectal tumors. There are three genes positively identifying mesenchymal tumor in the IHC test: HTR2B, FRMD6 and ZEB1. ZEB1 was not present on the arrays used and could thus not be evaluated. HTR2B and FRMD6 were both expressed to very low levels in this cohort (2log values lower than 4), for which we have no logical explanation. Indeed, in the CMS-3232 data set we observed a strong positive correlation between the chemotherapy-induced gene set and ZEB1, HTR2B and FRMD6, as expected, with ANOVA *P*-values of 3.2e^−193^, 6.8e^−149^ and 8.8e^−315^, respectively.

In this report we show that neoadjuvant therapy can influence molecular classification of primary colorectal tumors and liver metastases. Chemotherapy induces a shift toward a more mesenchymal-like phenotype. These results are in line with previous reports showing the treatment-resistant nature of mesenchymal-like tumors both in colon cancer^14, 29, 30^ and other types of cancer.^31^ Although chemotherapy has prolonged median overall survival of patients with metastatic CRC from ~6 months to over 2 years, tumor recurrence (almost) always ensues.^15, 32^ An in-depth analysis of the phenotype of residual tumor cells following chemotherapy will provide novel targets for therapy targeting residual disease. The results in the present study suggest that CMS4-targeted therapy may not only be effective in the treatment of CMS4-diagnosed tumors, but also in the adjuvant treatment of chemotherapy-surviving tumor residue of other CRC subtypes that gained a mesenchymal-like phenotype.

## Figures and Tables

**Table 1:** Patient characteristics

	*All tumors*		*Epithelial-like*		*Mesenchymal-like*		P*-value*
	n*=129*	*(%)*	n*=61*	*(%)*	n*=68*	*(%)*	
Age	61.9	(37–83)	62.4	(42–83)	61.5	(37–82)	
							
*Sex*							
Female	43	33.3	21	34.4	22	32.4	0.835
Male	86	66.7	40	65.6	46	67.6	
							
*Onset of metastasis*							
Synchronous	76	58.9	37	60.7	39	57.4	0.703
Metachronous	53	41.1	24	39.3	29	42.6	
							
*Site of primary tumor*							
Right colon	28	21.7	11	18	17	25	0.119
Transverse colon	4	3.1	0	0	4	5.9	
Left colon	13	10.1	7	11.5	6	8.8	
Sigmoid	39	30.2	25	41	14	20.6	
Rectosigmoid	4	3.1	1	1.6	2	2.9	
Rectum	41	31.8	16	26.2	25	36.8	
Unknown	1	0.8	1	1.6	0	0	
							
*Invasion depth*							
T2	9	7	6	9.8	3	4.4	0.506
T3	96	74.4	45	73.8	51	75	
T4	22	17.3	10	16.4	12	17.6	
Unknown	2	1.6	0	0	2	2.9	
							
*Lymph node status*							
N0	49	38	28	45.9	21	30.9	0.206
N1	48	37.2	21	34.4	27	39.7	
N2	29	22.5	11	18	18	26.5	
Unknown	3	2.3	1	1.6	2	2.9	
							
*Dukes classification*							
B1	5	3.9	3	4.9	2	2.9	0.733
B2	20	15.5	8	13.1	12	17.6	
C1	1	0.8	0	0	1	1.5	
C2	27	20.9	14	23	13	19.1	
D	75	58.1	36	59	39	57.4	
Unknown	1	0.8	0	0	1	1.5	
							
*Differentiation*							
Well	7	5.4	2	3.3	5	7.4	0.752
Well–moderate	2	1.6	1	1.6	1	1.5	
Moderate	78	60.5	37	60.7	41	60.3	
Moderate–poor	8	6.2	3	4.9	5	7.4	
Poor	11	8.5	7	11.5	4	5.9	
Unknown	23	17.9	11	18	11	16.2	
							
*Neoadjuvant radiotherapy*							
Yes	32	24.8	9	14.8	23	33.8	0.009
No	95	73.6	52	85.2	43	63.2	
							
*Neoadjuvant chemotherapy*							
Yes	13	10.1	2	3.3	11	16.2	0.013
No	114	88.4	59	96.7	55	80.9	
Unknown	2	1.6	0	0	2	2.9	
							
*Adjuvant chemotherapy*							
Yes	45	34.9	20	32.8	25	36.8	0.506
No	81	62.8	41	67.2	40	58.8	
Unknown	3	2.3	0	0	3	4.4	

Patient characteristics of the classified patients. Age was compared via the Wilcoxon rank sum test, for all other variables the *X*^2^-test was used.
