# Supplementary material for: Neoadjuvant chemotherapy affects molecular classification of colorectal tumors
Source: Oncogenesis. 2017 Jul 10;6(7):e357–. doi: 10.1038/oncsis.2017.48 (PMC5541704; doi:10.1038/oncsis.2017.48)
Supplement: Supplementary Table 1 [file oncsis201748x1.pdf]

Supplemental Table 1

Primary tumor - Liver metastatis	Concordant n=71	epithelial - mesenchymal n=25	mesenchymal - epithelial n=33	p-value
**Chemotherapy before liversurgery**				
yes	11	10	8	0,04
no	60	15	25	
**Neoadjuvant chemotherapy**				
yes	5	1	7	0,044
no	66	24	26	
**Neoadjuvant Radiotherapy**				
yes	21	2	9	0,093
no	50	23	24	
**Adjuvant chemotherapy**				
yes	9	3	7	0,475
no	62	22	26	
