# Supplementary material for: Neoadjuvant chemotherapy affects molecular classification of colorectal tumors
Source: Oncogenesis. 2017 Jul 10;6(7):e357–. doi: 10.1038/oncsis.2017.48 (PMC5541704; doi:10.1038/oncsis.2017.48)
Supplement: Supplementary Table Legends [file oncsis201748x4.docx]

**Supplemental Table 1 The influence of (neo-) adjuvant therapy on the classification of paired tumors**

**Supplemental** **Table 2 Gene expression profiling after 5-FU treatment in vitro**

**Supplemental Table 3 Gene expression profiles after chemotherapy treatment in patients** **compared to gene expression profiles after 5-FU treatment in vitro.**
